# Quantitative 0.55 T MRI of the Infraclavicular Brachial Plexus: Topography and Neurovascular Morphometry Relevant to Infraclavicular Block

**DOI:** 10.3390/diagnostics16193247

**Published:** 2026-10-08

**Authors:** Petar-Preslav Petrov, Delyan Dimitrov, Tanya Kitova, Pavel Timonov, Antoaneta Fasova, Darina Barbutska, Nikoleta Dimitrova, Yasen Getsov, Milena Sandeva

**Affiliations:** 1 Department of Anatomy, Histology and Embryology, Faculty of Medicine, Medical University of Plovdiv, 4002 Plovdiv, Bulgaria; 2 Faculty of Medicine, Medical University of Plovdiv, 4002 Plovdiv, Bulgaria; 3 Medical Simulation Training Centre, Faculty of Medicine, Medical University of Plovdiv, 4002 Plovdiv, Bulgaria; 4 Department of Forensic Medicine and Deontology, Faculty of Medicine, Medical University of Plovdiv, 4002 Plovdiv, Bulgaria; 5 Department of Occupational Diseases Including Clinical Allergology Activities, University Multiprofile Hospital for Active Treatment “St. George”, 4002 Plovdiv, Bulgaria; 6 Department of Occupational Diseases, Clinical Allergology and Toxicology, Faculty of Medicine, Medical University of Plovdiv, 4002 Plovdiv, Bulgaria; 7 Department of Anesthesiology and Intensive Care, Acibadem City Clinic University Hospital Vitosha, 1415 Sofia, Bulgaria; 8 Department of Midwifery Care, Faculty of Public Health, Medical University of Plovdiv, 4002 Plovdiv, Bulgaria

**Keywords:** brachial plexus, magnetic resonance imaging, clinical anatomy, infraclavicular block, axillary artery, axillary vein, morphometry, peripheral nervous system, regional anesthesia

## Abstract

**Background/Objectives:** Quantitative MRI data describing the infraclavicular brachial plexus cords together with both axillary vessels remain limited. This study characterized artery-centered cord topography and neurovascular morphometry on 0.55 T MRI and discussed the potential anatomical implications for infraclavicular block. **Methods:** This prospective cross-sectional imaging study included 100 adults undergoing clinically indicated MRI. One infraclavicular region was analyzed per participant. Within the mid-infraclavicular retropectoral region posterior to the pectoralis minor muscle, the axial T1-weighted image providing the clearest simultaneous visualization of the lateral cord (LC), medial cord (MC), posterior cord (PC), axillary artery (AA), and axillary vein (AV) was selected. The LC, MC, PC, and AV were mapped using a 12-sector clock-face model centered on the AA, and center-to-center intercord, cord-to-AA, and cord-to-AV distances were measured. **Results:** The LC was most frequently located at 11 o’clock (62%), the MC at 3 o’clock (41%), the PC at 6 o’clock (40%), and the AV at 2 o’clock (71.1%; n = 90). Mean intercord distances were 11.73 ± 3.17 mm (LC-MC), 11.09 ± 2.43 mm (LC-PC), and 8.13 ± 2.83 mm (MC-PC). Mean cord-to-AA distances were 6.33 ± 1.69, 7.36 ± 2.03, and 7.13 ± 1.83 mm for LC, MC, and PC, respectively. In 92 participants with complete cord-to-AV measurements, corresponding means were 17.01 ± 5.01, 9.61 ± 2.90, and 16.54 ± 3.87 mm. **Conclusions:** At the mid-infraclavicular retropectoral level, 0.55 T MRI demonstrated a structured but non-equidistant neurovascular arrangement. These imaging-derived data provide quantitative anatomical reference information but are not procedural safety margins or predictors of block success.

## 1. Introduction

The infraclavicular portion of the brachial plexus is a clinically important component of the peripheral nervous system because the lateral, medial, and posterior cords are in close relation to the axillary artery and vein. These neurovascular relationships are relevant to cross-sectional image interpretation and form the anatomical basis of infraclavicular brachial plexus block, an established regional anesthetic technique for surgery and analgesia of the distal upper extremity. Contemporary ultrasound guidance permits real-time visualization of the neural structures, axillary artery, axillary vein, needle trajectory, and local-anesthetic spread; nevertheless, accurate performance remains dependent on detailed recognition of the regional neurovascular anatomy [1,2].

The axillary artery is commonly used as the principal reference structure for infraclavicular block, but the cords should not be regarded as fixed or symmetrically distributed points around the vessel. Ultrasound studies have demonstrated preferential cord positions together with substantial interindividual variation. Kumar et al. described a 12-sector artery-centered model in which the lateral cord was most frequently found in sectors 10–11, the medial cord in sectors 2–4, and the posterior cord in sectors 6–7 [3]. Di Filippo et al. likewise emphasized the variable sonographic identification of the cords in the infraclavicular fossa [4]. More recently, our group used the same artery-centered framework in a prospective multicenter ultrasonographic study to quantify bilateral cord topography, intercord distances, and cord-to-vessel relationships in an independent cohort; that cohort did not overlap with the participants in the present MRI study [5]. Such observations are clinically relevant because expected position may assist orientation, but cannot replace patient-specific visualization.

MRI has a particularly important historical relationship with infraclavicular block. Klaastad et al. developed the lateral sagittal infraclavicular technique using MRI-based anatomical measurements [6], and the resulting approach was subsequently evaluated clinically [7]. Sauter et al. used MRI in volunteers to identify the location closest to all three infraclavicular cords in a sagittal plane corresponding to the lateral sagittal approach, demonstrating that cross-sectional imaging can inform block-relevant anatomical concepts without itself serving as the real-time guidance modality [8].

Ultrasound and MRI provide complementary rather than interchangeable views of infraclavicular anatomy. Ultrasound offers dynamic, real-time, patient-specific visualization in the procedural position, including vascular compressibility, needle trajectory, and injectate spread, but image acquisition is operator- and plane-dependent and may be influenced by transducer pressure. MRI provides transducer-free cross-sectional tissue contrast and a wider static anatomical context, but it does not reproduce procedural dynamics and is not a real-time guidance modality. The present study used 0.55 T MRI because the qualifying clinically indicated examinations were acquired on the 0.55 T clinical system available at the study site; field strength was not selected as a comparative experimental variable. Contemporary 0.55 T musculoskeletal MRI may offer practical and physical advantages such as wide-bore access and reduced susceptibility effects, while the lower signal-to-noise ratio relative to 1.5–3 T remains an important trade-off that requires field-strength-specific optimization [9]. Accordingly, differences between MRI- and ultrasound-derived infraclavicular measurements may reflect both interindividual anatomy and methodological factors, including imaging plane, anatomical level, limb position, transducer effects, and modality-specific visualization.

The anatomy encountered during infraclavicular block is also level-dependent. The costoclavicular approach targets the plexus more proximally, where the cords are typically grouped more compactly lateral to the axillary artery [10,11,12]. In addition, fascial layers and septa may influence injectate distribution within the infraclavicular neurovascular compartment [13,14]. These findings support a three-dimensional view of the region in which cord-to-cord and cord-to-vessel relationships may vary with the selected anatomical level and approach.

MRI and MR neurography provide complementary information regarding the course, morphology, and relationships of the brachial plexus [15,16,17,18,19]. Dedicated MR neurography is primarily optimized for nerve visualization and pathology, whereas quantitative MRI characterization of artery-centered cord and vessel relationships relevant to infraclavicular block has been less extensively studied. The present study therefore aimed to characterize, on 0.55 T MRI, (1) the clock-face topography of the LC, MC, PC, and AV relative to the AA; (2) intercord distances; (3) cord-to-AA distances; and (4) cord-to-AV distances. We hypothesized that the structures would demonstrate preferential population-level topographic clustering but a non-equidistant neurovascular configuration, without systematic right–left differences.

## 2. Materials and Methods

### 2.1. Study Design and Ethics

This was a prospective cross-sectional observational imaging study conducted at the Department of Diagnostic Imaging, Medico-Diagnostic Center ‘Medicus Alpha’, Plovdiv, Bulgaria. Participants were recruited and the MRI examinations included in the study were obtained between May and August 2026. MRI examinations were clinically indicated and were not performed solely for research purposes; the acquired images were additionally used for topographic and morphometric analysis of the infraclavicular region.

The design was descriptive and anatomical. It was not a diagnostic-accuracy study, a direct MRI-versus-ultrasound or field-strength comparison, or an interventional block study, and no procedural or clinical block outcomes were assessed.

The study formed part of an approved investigation of the infraclavicular region in human participants. The study was approved (Approval Code R-KNE-65/08.05.2026; Protocol No. 5/29.04.2026) by the Ethics Committee of Medical University of Plovdiv, Bulgaria, and was conducted in accordance with the Declaration of Helsinki. Written informed consent was obtained from all participants before inclusion.

### 2.2. Participants

Adult participants aged ≥18 years undergoing clinically indicated MRI examinations involving the shoulder and/or proximal humeral region were assessed for eligibility. Participants were included when the infraclavicular region was contained within the imaging field, an axial T1-weighted TSE series of sufficient quality was available, all predefined inclusion criteria were met, no exclusion criterion was present, and written informed consent had been provided. Additional inclusion requirements were reliable identification of the principal infraclavicular anatomical landmarks and preserved regional anatomy without traumatic, postoperative, neoplastic, inflammatory, or other pathological alterations capable of changing normal spatial relationships. Specific individual clinical indications were not included in the research dataset and are not reported under the approved study protocol.

Exclusion criteria were age <18 years, refusal or absence of informed consent, insufficient coverage of the infraclavicular region, absence of a suitable axial sequence, substantial motion or technical artifacts, previous trauma or surgery affecting the region, and pathological processes that altered the normal anatomy of the neural or vascular structures.

One infraclavicular region was analyzed per participant. The side corresponded to the clinically acquired examination; no contralateral examination was added solely for research purposes. The final dataset comprised 100 participants, including 50 right-sided and 50 left-sided examinations. This equal right–left distribution occurred by chance and was not imposed by a prespecified quota or balancing procedure. The target sample of 100 participants had been prespecified in the approved study design. Because the principal objectives were descriptive and morphometric, no formal a priori hypothesis-testing power calculation was performed.

### 2.3. MRI Equipment and Acquisition

MRI examinations were performed using a MAGNETOM Free.Max 0.55 T system (Siemens Healthineers, Erlangen, Germany). The morphometric analysis was based on a two-dimensional axial T1-weighted Turbo Spin Echo sequence. The acquisition parameters were: repetition time, 610 ms; echo time, 13 ms; slice thickness, 3.0 mm; slice spacing, 3.45 mm; two signal averages; echo train length, 3; nominal refocusing angle, 180 degrees; field of view, 160 × 160 mm; and approximate in-plane pixel dimensions, 0.357 × 0.357 mm. Images were acquired without intravenous contrast administration.

Participants were positioned head-first supine. The upper extremity on the examined side was positioned alongside the body without shoulder abduction. This position was maintained during acquisition of the axial series used for the topographic and morphometric analysis.

### 2.4. Image Review and Anatomical Identification

Image review and all study measurements were performed by a single study investigator, using RadiAnt DICOM Viewer 2026.1 (Medixant, Poznań, Poland). The LC, MC, PC, AA, and AV were identified using a combination of signal intensity, morphology, expected anatomical location, and spatial relationships. When necessary, structures were followed across adjacent axial slices to confirm their anatomical continuity and identity.

**Figure 1:**
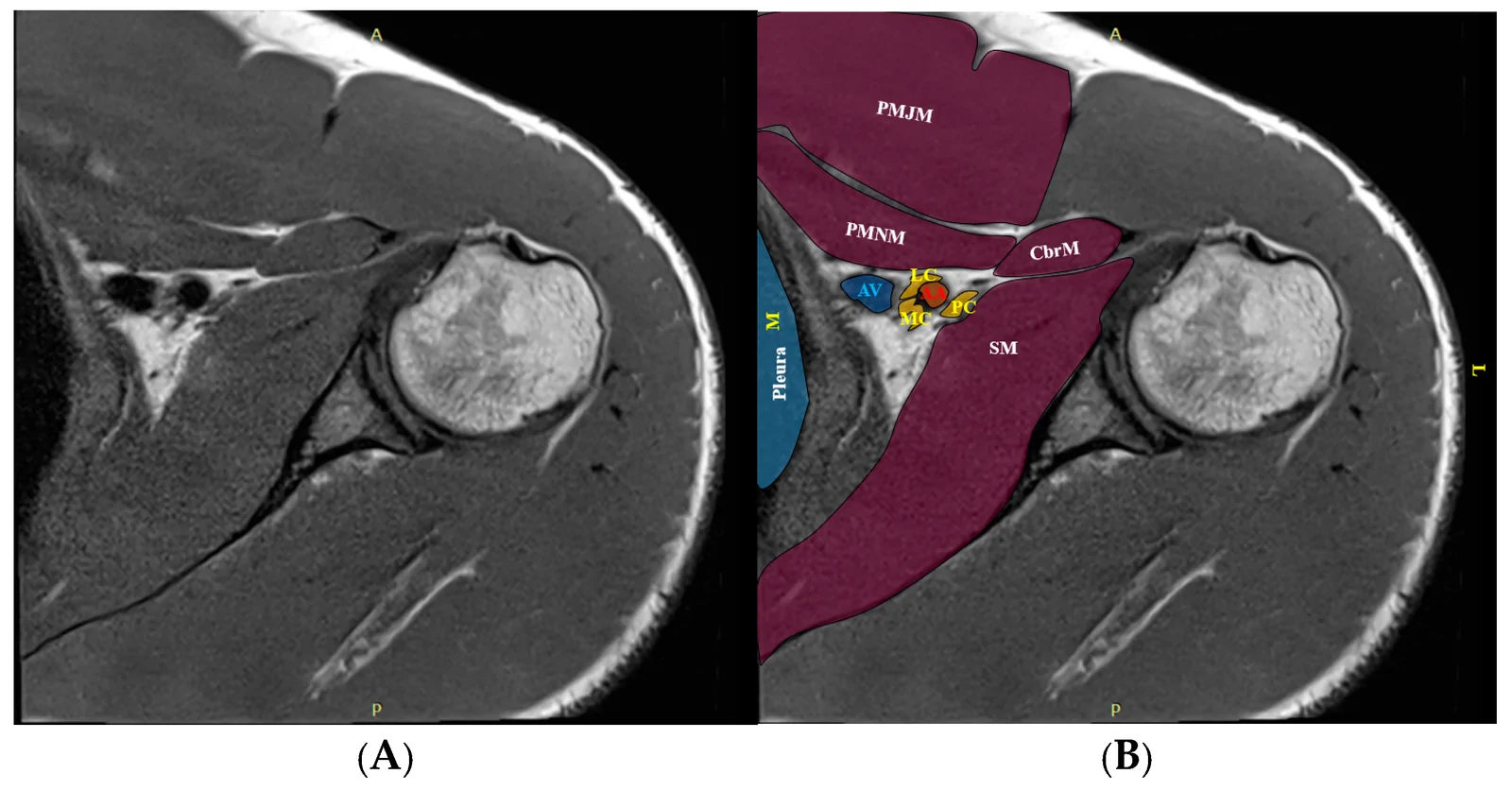
Representative axial T1-weighted MRI of the infraclavicular region. (**A**). Unannotated image. (**B**). Annotated image showing the principal neurovascular and muscular landmarks. Legend: lateral cord (LC), medial cord (MC), posterior cord (PC), axillary artery (AA), axillary vein (AV), pectoralis major muscle (PMJM), pectoralis minor muscle (PMNM), coracobrachialis muscle (CbrM), subscapularis muscle (SM), anterior (A), posterior (P), medial (M), and lateral (L).

On the T1-weighted sequence, perineural and perivascular fat provided intrinsic high-signal contrast against the relatively lower-signal neural, vascular, and muscular structures. The cords were identified as round or oval relatively low-signal neural structures within the perivascular fat. The AA was identified according to its morphology, location, and low intraluminal signal. The retropectoral position posterior to the pectoralis minor muscle was used as the principal anatomical landmark for defining the mid-infraclavicular analysis region (Figure 1).

### 2.5. Selection of the Axial Analysis Level

For each participant, quantitative analysis was performed within the mid-infraclavicular retropectoral region, posterior to the pectoralis minor muscle. Within this anatomically standardized region, the axial T1-weighted image providing the clearest simultaneous delineation of the LC, MC, PC, and adjacent axillary vessels was selected for quantitative analysis. Adjacent slices were reviewed when necessary to confirm structural continuity and identity. Selection of the single analysis slice therefore retained a reader-dependent component, but it was constrained to the predefined central retropectoral segment.

All measurements were therefore obtained within the same anatomically defined central retropectoral infraclavicular segment rather than at variable proximal or distal levels along the brachial plexus. Standardization was based on this regional anatomical criterion rather than on a fixed osseous coordinate; minor within-segment proximodistal variation was therefore possible.

### 2.6. Topographic Analysis

**Figure 2:**
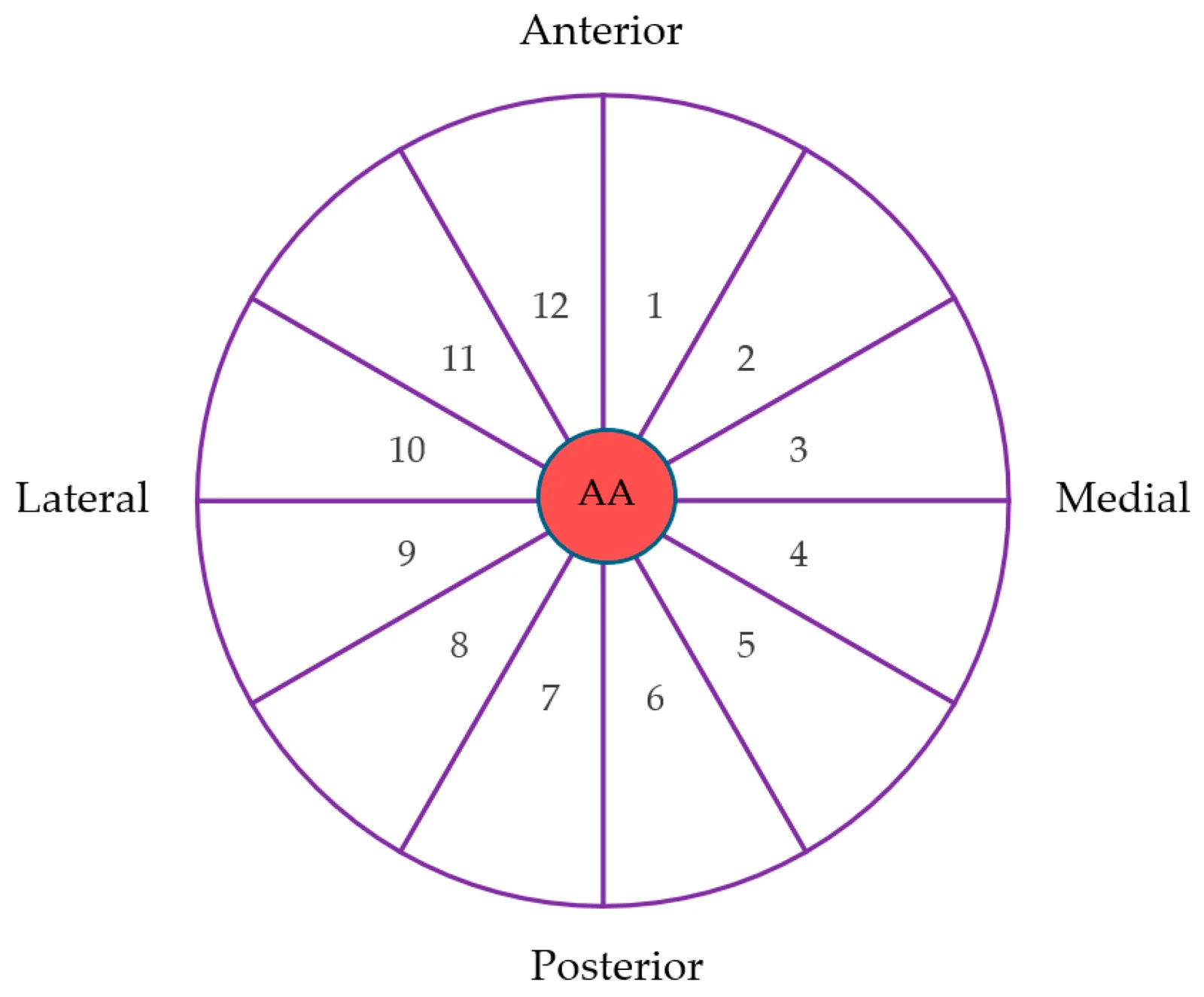
Standardized artery-centered 12-sector clock-face system used for MRI topographic analysis. The center of the axillary artery (AA) was defined as the origin; 12 o’clock corresponded to anterior, 3 o’clock to medial, 6 o’clock to posterior, and 9 o’clock to lateral. The same anatomically standardized orientation was applied to right- and left-sided examinations.

The AA was the sole central reference structure for the clock-face coordinate system. A standardized 12-sector model was applied, with 12 o’clock corresponding to anterior, 3 o’clock to medial, 6 o’clock to posterior, and 9 o’clock to lateral; the sectors represented mutually exclusive 30-degree angular intervals. The same anatomical orientation was applied to right- and left-sided examinations, independent of native radiological display orientation. The visually defined center of each LC, MC, PC, and AV cross-section was assigned to the single sector containing that center relative to the center of the AA. Thus, a structure whose outer contour crossed a sector boundary was not assigned to two sectors; classification was determined by its center. The AV did not define the coordinate system and was not used as a moving reference origin; rather, it was analyzed as a neighboring vascular structure with its own artery-centered sector position and cord-to-AV distances (Figure 2).

### 2.7. Morphometric Measurements

Morphometric measurements were performed on the selected axial image using the electronic calipers of RadiAnt DICOM Viewer. All linear distances were recorded in millimeters. A standardized center-to-center principle was used: the center of each structure was visually defined as the geometric midpoint of its visible cross-sectional outer contour on the selected axial image, and the distance between the relevant centers was measured. No automated segmentation or area-based centroid calculation was used. The nominal in-plane pixel dimensions were approximately 0.357 × 0.357 mm; this represents spatial sampling and should not be interpreted as an empirical measurement-error or repeatability estimate.

**Figure 3:**
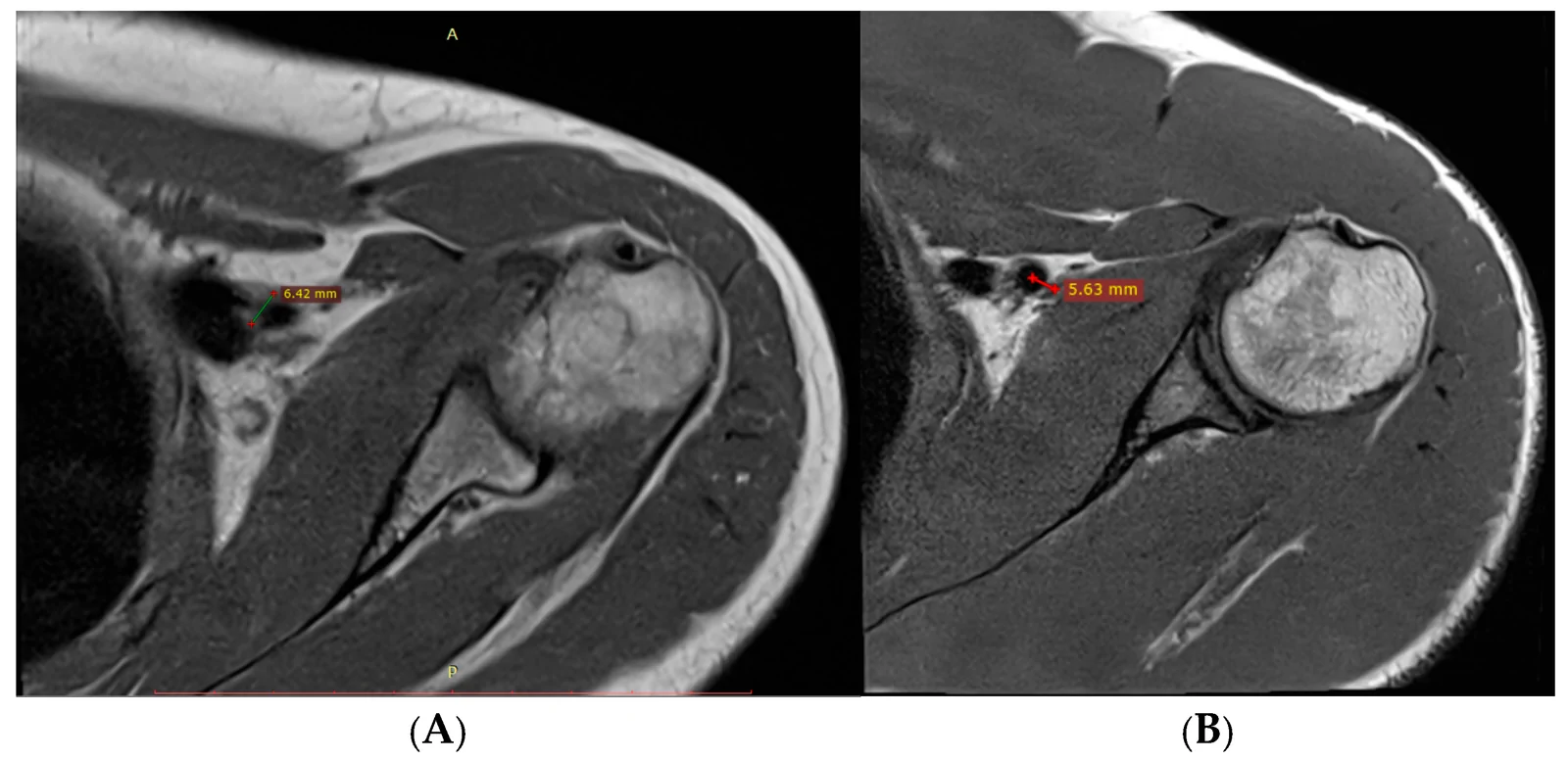
Representative center-to-center morphometric measurements on axial T1-weighted MRI: (**A**): lateral-to-medial cord distance. (**B**): posterior cord-to-axillary artery distance.

Three distance families were analyzed: (1) intercord distances: LC-MC, LC-PC, and MC-PC; (2) cord-to-AA distances: LC-AA, MC-AA, and PC-AA; and (3) cord-to-AV distances: LC-AV, MC-AV, and PC-AV; repeated measurements and averaging were not performed. Pleural distance measurements were not included in the present study. Representative center-to-center measurements are shown in Figure 3.

### 2.8. Structure-Specific Missing Data

If a structure could not be identified with sufficient confidence on the basis of its morphology, anatomical position, and continuity across adjacent images, the corresponding structure-specific topographic classification or morphometric measurement was recorded as missing. Other reliably determined observations from the same participant were retained. Missing values were not imputed. Topographic classification and complete distance-family evaluability were recorded separately and therefore could have different denominators.

### 2.9. Statistical Analysis

Data were organized using Microsoft Excel 2016 (Microsoft Corporation, Redmond, WA, USA). Statistical analyses were performed primarily with jamovi version 2.6.44 (The jamovi Project, Sydney, Australia); IBM SPSS Statistics version 19 (IBM Corp., Armonk, NY, USA) was used for selected additional procedures.

Continuous variables were summarized using the number of valid observations, mean and standard deviation (SD), 95% confidence interval (CI) for the mean, median and interquartile range (IQR), and minimum–maximum range. Categorical variables were summarized as counts and percentages with 95% CIs. Analyses were performed using available observations without imputation of structure-specific missing values.

Clock-face positions were additionally summarized using circular statistics. The circular mean position and mean resultant length (R) were calculated for each structure; values of R closer to 1 indicate greater concentration around the mean angular position. Where applicable, 95% CIs for circular means were obtained using bootstrap procedures. Because right- and left-sided MRI examinations represented independent participants, side differences in sector distributions were evaluated using chi-square statistics with Monte Carlo *p*-values. Holm correction was applied across the four anatomical structures.

Continuous morphometric outcomes were analyzed using three linear mixed-effects models: one for intercord distances, one for cord-to-AA distances, and one for cord-to-AV distances. Although each participant contributed only one side, each participant contributed up to three related measurements within a given distance family. The participant identifier was therefore entered as a random intercept to account for within-participant correlation among the repeated distance-type outcomes. Distance type, sex, side, and their interactions were included as fixed effects. Omnibus F-tests were followed, where appropriate, by estimated marginal means (EMMs) and pairwise comparisons. Holm correction was applied to multiple pairwise comparisons. In an exploratory post hoc sensitivity analysis requested to assess potential body-size confounding of sex effects, height was added as an additional covariate (main effect only) to each of the three mixed-effects models while retaining the original fixed-effect interaction structure and participant random intercept. Model convergence and residual diagnostics were assessed. All tests were two-sided, and *p* < 0.05 was considered statistically significant.

## 3. Results

### 3.1. Participant Characteristics and Evaluability

The MRI cohort included 100 participants (51 men and 49 women), with a mean age of 46.09 ± 17.11 years (range, 18–81 years). Fifty examinations were right-sided and 50 were left-sided. Additional anthropometric characteristics are presented in Table 1.

The LC, MC, PC, and AA were reliably identified for the planned cord and artery-centered analyses in all 100 examinations (100%). AV sector classification was available in 90/100 examinations (90.0%). Complete LC-AV, MC-AV, and PC-AV distance measurements were available in 92/100 participants (92.0%). All intercord and cord-to-AA measurements were available in the full cohort. Because topographic classification and morphometric evaluability were recorded separately, their structure-specific denominators were analyzed independently.

### 3.2. Topographic Distribution Around the Axillary Artery

The LC was most frequently located at 11 o’clock (62/100, 62.0%), followed by 12 o’clock (19.0%) and 10 o’clock (10.0%). The MC was most frequently located at 3 o’clock (41.0%), followed by 4 o’clock (34.0%) and 5 o’clock (17.0%). The PC was most frequently located at 6 o’clock (40.0%), followed by 5 o’clock (26.0%) and 7 o’clock (21.0%). Among the 90 examinations with AV sector classification, the AV was most frequently located at 2 o’clock (64/90, 71.1%), followed by 3 o’clock (23.3%) (Table 2). Detailed right- and left-sided sector distributions are provided in Table S1.

Circular analysis demonstrated marked clustering around characteristic positions. Overall circular mean positions were 11.19 o’clock for the LC (95% CI, 11.04–11.35; R = 0.901), 3.86 o’clock for the MC (95% CI, 3.67–4.05; R = 0.875), 5.84 o’clock for the PC (95% CI, 5.64–6.04; R = 0.865), and 2.27 o’clock for the AV (95% CI, 2.16–2.40; R = 0.953). Detailed circular characteristics overall and according to side are provided in Table S2.

The modal position was identical on the right and left for all four structures: 11 o’clock for the LC, 3 o’clock for the MC, 6 o’clock for the PC, and 2 o’clock for the AV. Monte Carlo chi-square comparisons did not demonstrate significant right–left differences after Holm correction for the LC (adjusted *p* = 0.5827), MC (adjusted *p* = 0.5827), PC (adjusted *p* = 1.0000), or AV (adjusted *p* = 1.0000). Detailed Monte Carlo chi-square comparisons are provided in Table S3.

### 3.3. Morphometric Relationships

The directly observed morphometric measurements are summarized in Table 3.

#### 3.3.1. Intercord Distances

**Figure 4:**
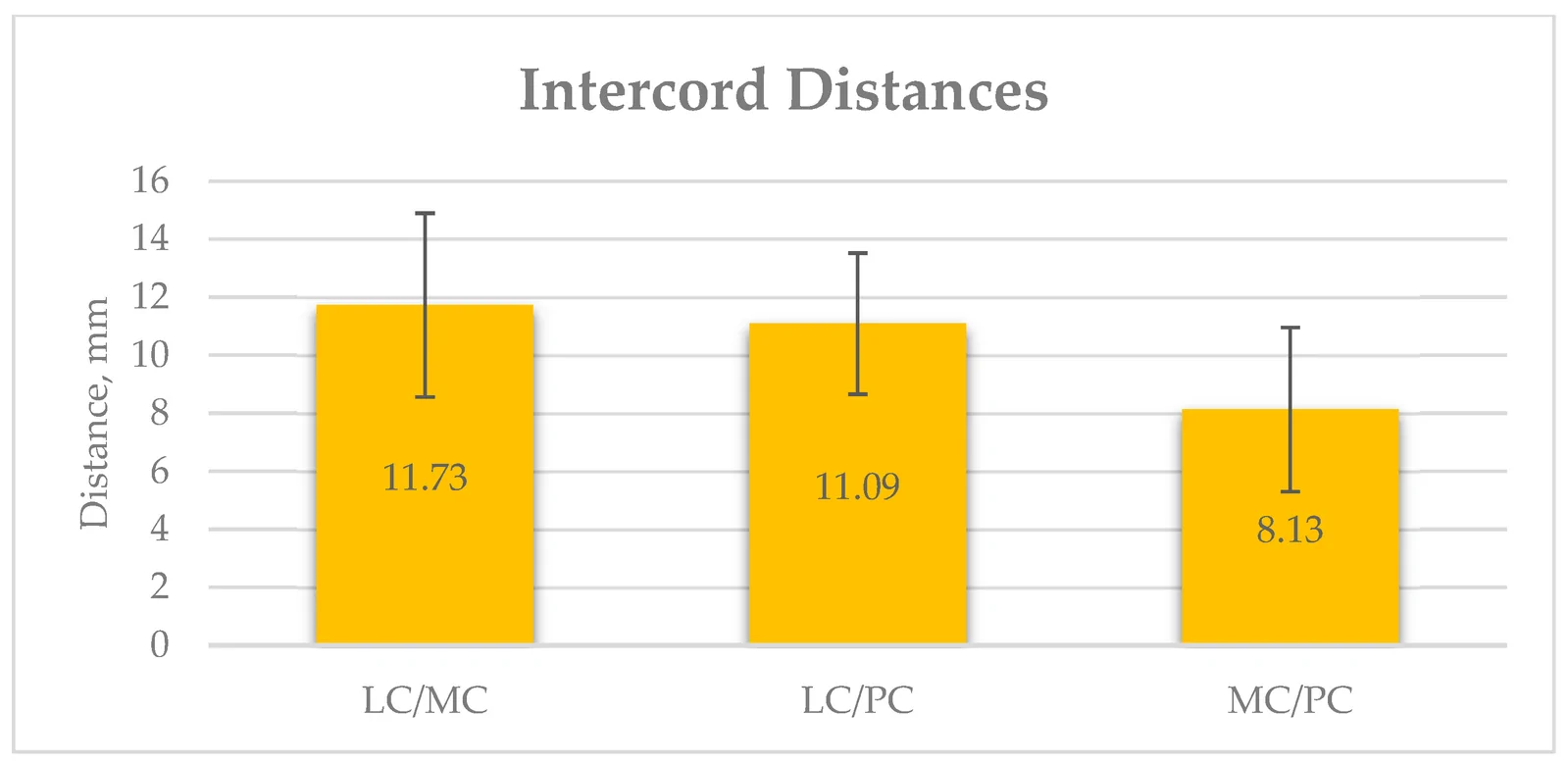
Intercord distances between the brachial plexus cords in the infraclavicular region. Values are presented as mean ± standard deviation.

The greatest observed mean intercord distance was LC-MC (11.73 ± 3.17 mm), followed by LC-PC (11.09 ± 2.43 mm), whereas MC-PC was the shortest (8.13 ± 2.83 mm). This is presented in Figure 4. The mixed-effects analysis included 300 distance observations from 100 participants and demonstrated a significant main effect of distance type, F(2,192) = 64.547, *p* < 0.0001. The overall effect of sex was also significant, F(1,96) = 6.864, *p* = 0.0102, whereas the effect of side did not reach statistical significance, F(1,96) = 3.505, *p* = 0.0642.

A significant distance type × sex interaction was observed, F(2,192) = 3.163, *p* = 0.0445. Model-based EMMs were 11.71 mm for LC-MC, 11.08 mm for LC-PC, and 8.13 mm for MC-PC. Holm-adjusted pairwise comparisons showed that MC-PC was significantly shorter than LC-MC (difference, 3.583 mm; adjusted *p* < 0.0001) and LC-PC (difference, 2.954 mm; adjusted *p* < 0.0001), whereas LC-MC and LC-PC did not differ significantly after correction (adjusted *p* = 0.0632). The sex interaction was principally attributable to LC-MC, for which the model-estimated difference between men and women was +1.994 mm (Holm-adjusted *p* approximately 0.0012).

Descriptive intercord distances according to side and sex are provided in Table S4, and the detailed mixed-effects model results are provided in Table S5.

#### 3.3.2. Distances from the Cords to the Axillary Artery

**Figure 5:**
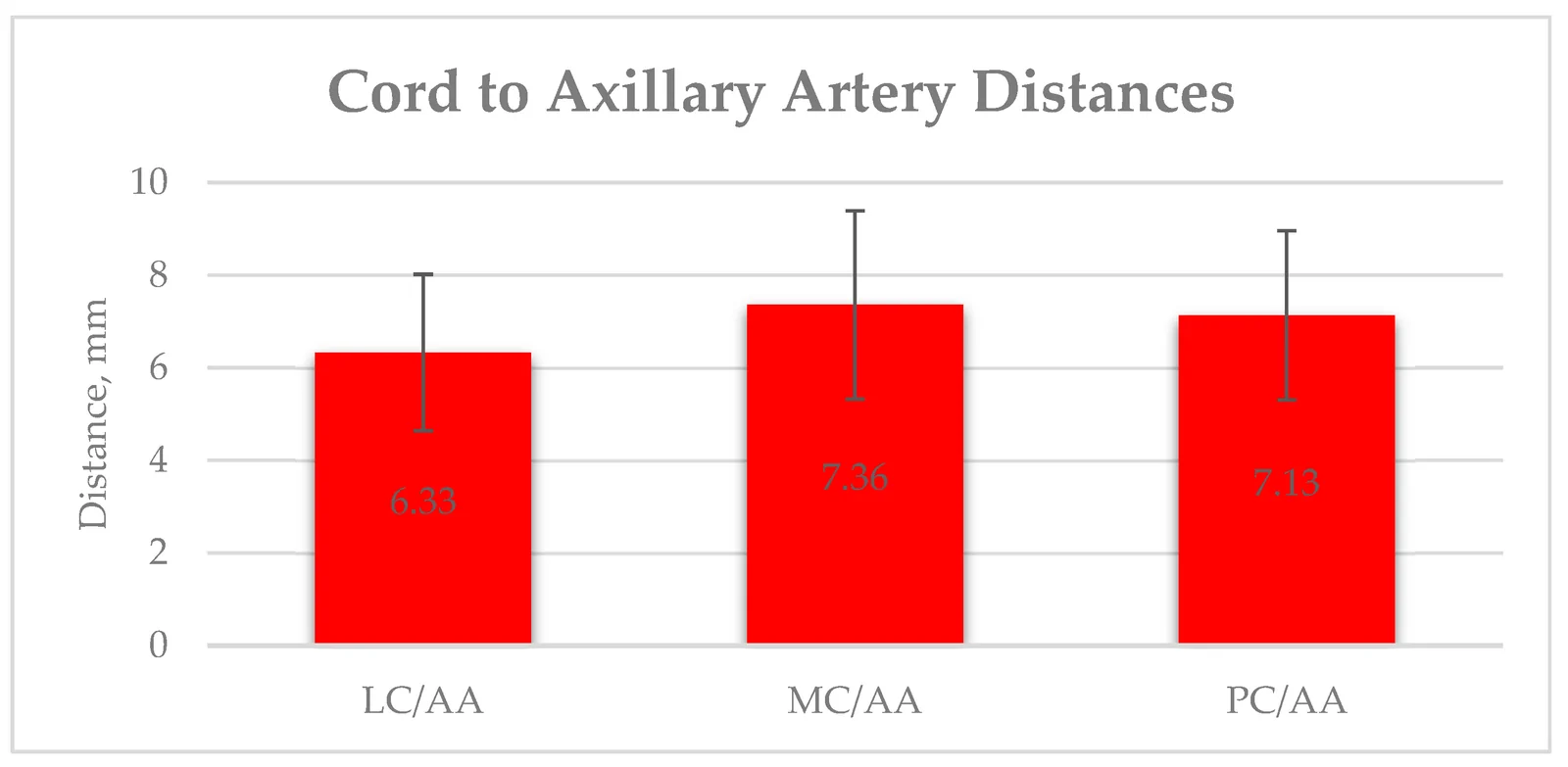
Cord-to-axillary artery distances in the infraclavicular region. Values are presented as mean ± standard deviation.

Observed mean cord-to-AA distances were 6.33 ± 1.69 mm for LC-AA, 7.36 ± 2.03 mm for MC-AA, and 7.13 ± 1.83 mm for PC-AA. This is presented in Figure 5. The mixed-effects analysis included 300 observations from 100 participants and identified a significant main effect of distance type, F(2,192) = 13.449, *p* < 0.0001, and a significant overall effect of sex, F(1,96) = 11.844, *p* = 0.0009. No significant overall side effect was observed, F(1,96) = 0.087, *p* = 0.7682, and none of the investigated interactions were significant.

Model-based EMMs were 6.32 mm for LC-AA, 7.35 mm for MC-AA, and 7.13 mm for PC-AA. LC-AA was significantly shorter than MC-AA (difference, −1.031 mm; Holm-adjusted *p* < 0.0001) and PC-AA (difference, −0.804 mm; adjusted *p* = 0.0003), whereas MC-AA and PC-AA did not differ significantly (adjusted *p* = 0.2789). Across the distance family, the model-estimated difference between men and women was +0.936 mm (95% CI, 0.401–1.471; *p* = 0.0009).

Detailed results of the cord-to-AA mixed-effects model are provided in Table S6.

#### 3.3.3. Distances from the Cords to the Axillary Vein

**Figure 6:**
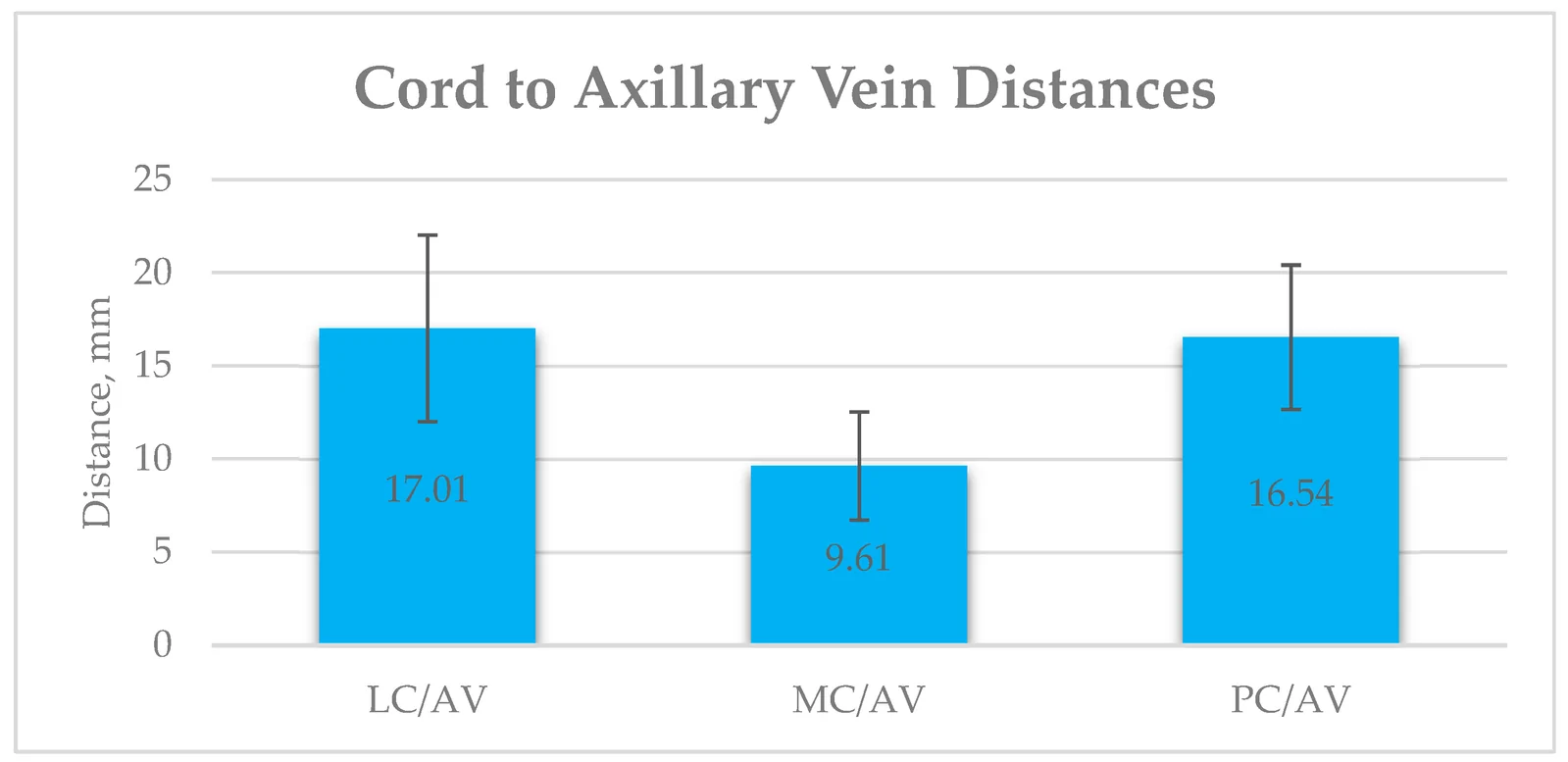
Cord-to-axillary vein distances in the infraclavicular region. Values are presented as mean ± standard deviation.

Complete cord-to-AV measurements were available in 92 participants, yielding 276 distance observations. Mean distances were 17.01 ± 5.01 mm for LC-AV, 9.61 ± 2.90 mm for MC-AV, and 16.54 ± 3.87 mm for PC-AV (Figure 6). The mixed-effects model demonstrated a strong main effect of distance type, F(2,176) = 198.695, *p* < 0.0001, and a significant overall effect of sex, F(1,88) = 6.476, *p* = 0.0127. The overall effect of side was not significant, F(1,88) = 1.122, *p* = 0.2923, and no interaction was statistically significant.

Model-based EMMs were 17.04 mm for LC-AV, 9.62 mm for MC-AV, and 16.55 mm for PC-AV. MC-AV was significantly shorter than both LC-AV and PC-AV (both Holm-adjusted *p* < 0.0001). LC-AV and PC-AV did not differ significantly (adjusted *p* = 0.2408). Thus, the model-supported pattern was a shorter MC-AV distance, with similar LC-AV and PC-AV distances. The principal fixed effects from the three linear mixed-effects models are summarized in Table 4.

All other investigated two-way and three-way interactions were non-significant. Pairwise comparisons were Holm-adjusted.

Detailed descriptive and mixed-effects results for cord-to-AV distances are provided in Table S7.

#### 3.3.4. Exploratory Height-Adjusted Sensitivity Analysis

Exploratory height-adjusted sensitivity analyses attenuated the sex effects. For intercord distances, height was significant, F(1,95) = 11.882, *p* = 0.0008, whereas the overall sex effect was no longer significant, F(1,95) = 1.058, *p* = 0.3063; the distance type × sex interaction remained significant, F(2,192) = 3.163, *p* = 0.0445. For cord-to-AA distances, both height, F(1,95) = 5.652, *p* = 0.0194, and sex, F(1,95) = 4.717, *p* = 0.0323, remained significant, although the sex effect was attenuated relative to the primary model. For cord-to-AV distances, neither height, F(1,87) = 2.980, *p* = 0.0878, nor sex, F(1,87) = 2.176, *p* = 0.1438, was significant after adjustment. Side remained non-significant in all three height-adjusted models. These analyses were exploratory and did not replace the prespecified primary models.

Detailed results of the exploratory height-adjusted sensitivity analyses are provided in Table S8.

## 4. Discussion

This study provides quantitative imaging-defined reference anatomy of the infraclavicular brachial plexus and axillary vessels at 0.55 T. The principal findings were fourfold: (1) the LC, MC, PC, and AV demonstrated distinct preferential clock-face positions around the AA; (2) the cords formed a non-equidistant configuration, with MC-PC representing the shortest intercord distance; (3) the LC was the closest cord to the AA at the analyzed axial level; and (4) the MC was substantially closer to the AV than either the LC or PC. No systematic right–left differences were identified in the principal topographic or morphometric analyses. Together, these findings characterize the normal neurovascular geometry of the mid-infraclavicular retropectoral region and provide an imaging-based anatomical framework relevant to infraclavicular block.

The artery-centered topographic pattern was concentrated but not fixed. The LC clustered predominantly in the anterior to anterolateral region, the MC in the medial to posteromedial region, and the PC in the posterior region. This broad organization is compatible with previous ultrasound studies showing preferential cord positions around the AA while also demonstrating meaningful interindividual variation [3,4]. The high resultant lengths in the present circular analysis indicate that variability should not be equated with random arrangement: each structure had a characteristic population-level distribution, but adjacent-sector dispersion may be relevant to image interpretation. For infraclavicular block, such population-level expectations may assist orientation, but they should not substitute for direct identification of neural and vascular structures in the individual patient.

Comparison with our previously published ultrasonographic study is informative because the two investigations used independent, non-overlapping cohorts and different acquisition conditions [5]. The broad artery-centered distribution of the three cords was concordant at a population level, but some vessel-centered relationships differed: the MC was closest to the AA in the ultrasound cohort, whereas the LC was closest in the present MRI cohort. These observations are not a head-to-head modality comparison. Ultrasound and MRI differ in acquisition geometry and visualization, and the studies also differed in participant samples and limb position (90-degree arm abduction in the ultrasound study versus the arm alongside the body on MRI). Arm abduction itself can alter infraclavicular relationships [20]. The between-study differences may therefore reflect a combination of interindividual anatomical variation and methodological factors rather than either explanation alone.

The present findings also extend earlier MRI work that linked cross-sectional anatomy to infraclavicular block design. Klaastad et al. used MRI to develop the lateral sagittal technique [6], and Sauter et al. used MRI to identify a location in a sagittal block-relevant plane that was close to all three cords [8]. The current study differs in objective and imaging plane: rather than selecting an injection point, it quantified artery-centered axial topography and three families of center-to-center distances within a predefined mid-infraclavicular retropectoral segment. The finding that the LC was closest to the AA should therefore be interpreted within this specific axial level and not as a contradiction of prior MRI-derived block concepts.

The intercord measurements demonstrate that the cords do not form an equilateral arrangement. MC-PC was approximately 3 mm shorter than either distance involving the LC after model adjustment. This finding has potential anatomical relevance to local-anesthetic distribution because a single perivascular deposit does not encounter three targets separated by equivalent distances. However, distance alone cannot determine injectate spread. [21] Cadaveric work has shown that fascial layers can restrict or redirect injectate during infraclavicular block [13], and microanatomical studies have demonstrated septal organization between the LC and the MC/PC complex in the costoclavicular region [14]. Therefore, the present morphometry should be considered a geometric substrate for block anatomy rather than a direct predictor of local-anesthetic coverage.

The cord-to-AA analysis is similarly relevant to the use of the AA as the principal landmark in many infraclavicular techniques. At the optimally visualized axial MRI level, the LC was significantly closer to the arterial center than the MC or PC. This observation emphasizes that the AA is a reference structure within an asymmetric neural configuration rather than the center of three equally distant cords. Anatomical variability of the brachial plexus is well established [22], and cord nomenclature should not be interpreted as implying a constant cross-sectional position throughout the entire infraclavicular course.

The relationship to the AV may also be anatomically relevant. The MC had the shortest center-to-center distance to the AV, whereas LC-AV and PC-AV were substantially larger and statistically similar. Axillary venous anatomy is variable and does not simply mirror the arterial pattern [23]. The AV is also more susceptible than the artery to changes in caliber, shape, and position. For this reason, the AV was treated in the present analysis as a measured neighboring structure rather than as the origin of the coordinate system. Center-to-center cord-to-AV measurements are not direct vascular safety margins: actual needle clearance depends on neural dimensions, vessel caliber, edge-to-edge separation, needle trajectory, physiologic state, and tissue deformation.

The selected anatomical level requires explicit consideration. More proximal costoclavicular approaches target the cords where they are generally more compactly grouped lateral to the AA [10,11,12], whereas conventional or lateral sagittal infraclavicular approaches encounter a more dispersed perivascular configuration. The comparative anatomical and clinical literature confirms that different infraclavicular approaches traverse different spatial corridors [24,25]. In addition, interindividual variation at the roots, trunks, divisions, or more proximal cord-formation levels cannot be inferred from a single distal cross-sectional level [22]. The present findings therefore apply specifically to the mid-infraclavicular retropectoral segment posterior to the pectoralis minor muscle and should not be generalized to the more proximal costoclavicular or more distal axillary portions of the plexus.

No systematic right–left difference was demonstrated. The same modal sector was observed on both sides for each structure, and side was not a significant main effect in any of the three morphometric models. These findings do not establish absolute bilateral equivalence, because different participants contributed right- and left-sided observations, but they provide no evidence of a consistent directional asymmetry in this cohort.

Sex-related effects in the primary models required cautious interpretation because absolute anatomical distances may scale with body size. The exploratory height-adjusted sensitivity analyses supported this concern. After adjustment for height, the overall sex effect was no longer significant for intercord or cord-to-AV distances, whereas it remained significant but was attenuated for cord-to-AA distances. Height itself was significantly associated with intercord and cord-to-AA distances. The significant distance type × sex interaction in the intercord family persisted after height adjustment. These findings suggest that body size accounts for part, but not necessarily all, of the observed sex-related variation and caution against attributing differences in absolute morphometric distances to sex alone.

A distinctive feature of the present study is the use of a modern 0.55 T clinical MRI system. The study leveraged routine clinically indicated examinations acquired on this platform rather than selecting field strength as an experimental comparison. Modern low-field musculoskeletal MRI can provide diagnostically useful anatomical imaging and may benefit from reduced magnetic susceptibility effects and wide-bore system design, but lower signal-to-noise ratio relative to 1.5–3 T remains an inherent trade-off [9]. In the present cohort, routine axial T1-weighted imaging provided sufficient fat-neural and fat-vascular contrast for the planned measurements in included examinations. However, the protocol was not a dedicated MR neurography protocol, and the study was not designed to establish diagnostic equivalence to higher-field MRI or to evaluate MR neurography performance [16,17,18,19].

Several additional limitations should be acknowledged. First, the analysis level was anatomically standardized to the mid-infraclavicular retropectoral region posterior to the pectoralis minor muscle rather than to a fixed osseous coordinate, and selection of the clearest single axial slice retained a reader-dependent component; minor within-segment proximodistal variation cannot be excluded. Second, only one side was analyzed per participant, preventing paired within-subject assessment of bilateral symmetry. Third, all measurements were performed by a single study investigator, and formal intraobserver and interobserver reproducibility was not assessed. No empirical ICC or repeatability-derived measurement-error estimate is available, and the nominal pixel dimensions should not be interpreted as such an estimate. Fourth, although the cords and AA were evaluable in all included examinations, AV topographic and morphometric evaluability was incomplete, and structure-specific missing observations were analyzed without imputation. Fifth, the exploratory height-adjusted analyses were post hoc and should be interpreted as sensitivity analyses rather than replacement primary models. Sixth, the study was anatomical rather than interventional: needle trajectory, edge-to-edge clearance, local-anesthetic spread, block onset, success, and complications were not evaluated. The reported distances are therefore imaging-derived anatomical measurements and require prospective procedural validation before any inference regarding block safety or efficacy.

## 5. Conclusions

At the mid-infraclavicular retropectoral level posterior to the pectoralis minor muscle, 0.55 T MRI demonstrated a structured but non-equidistant arrangement of the brachial plexus cords and axillary vessels. The LC was most commonly located at 11 o’clock, the MC at 3 o’clock, the PC at 6 o’clock, and the AV at 2 o’clock relative to the AA. MC-PC was the shortest intercord interval, the LC was closest to the AA, and the MC was closest to the AV. No systematic right–left effect was identified.

These findings provide quantitative imaging-derived anatomical reference data specifically for the mid-infraclavicular retropectoral neurovascular region. They reinforce that infraclavicular brachial plexus anatomy is three-dimensional, variable, and level-dependent rather than a set of fixed clock-face coordinates. Because the measurements were center-to-center and no block procedures or clinical outcomes were assessed, the results should not be translated directly into procedural safety margins, needle targets, or predictions of block success without prospective validation.

## Figures and Tables

**Table 1:** Participant characteristics.

Characteristic	Overall
Participants, n	100
Men, n (%)	51 (51.0)
Women, n (%)	49 (49.0)
Age, years	46.09 ± 17.11 (18–81)
Height, m	1.747 ± 0.092
Body weight, kg	86.39 ± 22.53
BMI, kg/m^2^	28.25 ± 6.98
Right-sided examinations, n (%)	50 (50.0)
Left-sided examinations, n (%)	50 (50.0)

Continuous values are presented as mean ± SD; age is additionally shown as range.

**Table 2:** Topographic distribution of the brachial plexus cords and axillary vein relative to the axillary artery.

Structure	Clock Sector	n/N	%	95% CI
LC	1	5/100	5.0	2.2–11.2
LC	2	2/100	2.0	0.6–7.0
LC	7	1/100	1.0	0.2–5.4
LC	9	1/100	1.0	0.2–5.4
LC	10	10/100	10.0	5.5–17.4
LC	11	62/100	62.0	52.2–70.9
LC	12	19/100	19.0	12.5–27.8
MC	2	1/100	1.0	0.2–5.4
MC	3	41/100	41.0	31.9–50.8
MC	4	34/100	34.0	25.5–43.7
MC	5	17/100	17.0	10.9–25.5
MC	6	5/100	5.0	2.2–11.2
MC	7	2/100	2.0	0.6–7.0
PC	3	2/100	2.0	0.6–7.0
PC	4	7/100	7.0	3.4–13.7
PC	5	26/100	26.0	18.4–35.4
PC	6	40/100	40.0	30.9–49.8
PC	7	21/100	21.0	14.2–30.0
PC	8	4/100	4.0	1.6–9.8
AV	1	2/90	2.2	0.6–7.7
AV	2	64/90	71.1	61.0–79.5
AV	3	21/90	23.3	15.8–33.1
AV	4	2/90	2.2	0.6–7.7
AV	5	1/90	1.1	0.2–6.0

LC, lateral cord; MC, medial cord; PC, posterior cord; AV, axillary vein.

**Table 3:** Observed morphometric measurements.

Family	Measurement	N	Mean ± SD, mm	95% CI, mm	Median (IQR), mm	Min–Max, mm
Intercord	LC-MC	100	11.73 ± 3.17	11.10–12.36	11.68 (4.18)	4.90–21.85
Intercord	LC-PC	100	11.09 ± 2.43	10.61–11.57	11.09 (3.48)	5.61–16.82
Intercord	MC-PC	100	8.13 ± 2.83	7.57–8.70	7.47 (3.09)	4.05–22.72
Cord-to-AA	LC-AA	100	6.33 ± 1.69	6.00–6.67	6.08 (1.94)	2.89–12.15
Cord-to-AA	MC-AA	100	7.36 ± 2.03	6.96–7.77	6.96 (2.91)	4.19–13.51
Cord-to-AA	PC-AA	100	7.13 ± 1.83	6.77–7.50	7.16 (2.26)	3.85–15.40
Cord-to-AV	LC-AV	92	17.01 ± 5.01	15.97–18.04	16.36 (7.52)	2.15–28.12
Cord-to-AV	MC-AV	92	9.61 ± 2.90	9.01–10.21	9.49 (3.76)	4.44–19.47
Cord-to-AV	PC-AV	92	16.54 ± 3.87	15.74–17.34	16.43 (5.26)	8.61–28.60

AA, axillary artery; AV, axillary vein; LC, lateral cord; MC, medial cord; PC, posterior cord. All values are single center-to-center measurements; no repeatability-derived measurement-error estimate was available.

**Table 4:** Principal fixed effects from the three linear mixed-effects models.

Distance Family	Observations/ Participants	Distance Type	Sex	Side	Significant Interaction
Intercord	300/100	F(2,192) = 64.547; *p* < 0.0001	F(1,96) = 6.864; *p* = 0.0102	F(1,96) = 3.505; *p* = 0.0642	Distance type × sex: F(2,192) = 3.163; *p* = 0.0445
Cord-to-AA	300/100	F(2,192) = 13.449; *p* < 0.0001	F(1,96) = 11.844; *p* = 0.0009	F(1,96) = 0.087; *p* = 0.7682	None significant
Cord-to-AV	276/92	F(2,176) = 198.695; *p* < 0.0001	F(1,88) = 6.476; *p* = 0.0127	F(1,88) = 1.122; *p* = 0.2923	None significant

## Data Availability

Participant-level data are not publicly available because of privacy and ethical restrictions. Requests for access may be considered by the corresponding author subject to applicable ethics approval and institutional requirements.

## Supplementary Materials

The following supporting information can be downloaded at: https://www.mdpi.com/article/10.3390/diagnostics16193247/s1**:** Table S1, right- and left-sided topographic distributions of the LC, MC, PC, and AV; Table S2, circular characteristics overall and according to side; Table S3, Monte Carlo chi-square comparisons of right- and left-sided sector distributions; Table S4, descriptive intercord distances according to side and sex; Table S5, detailed linear mixed-effects analysis of intercord distances; Table S6, detailed linear mixed-effects analysis of cord-to-AA distances; Table S7, detailed linear mixed-effects analysis of cord-to-AV distances; Table S8, exploratory height-adjusted sensitivity analyses of the three distance families.

## Abbreviations

The following abbreviations are used in this manuscript:

AA	Axillary artery
AV	Axillary vein
LC	Lateral cord
MC	Medial cord
PC	Posterior cord
BMI	Body mass index
CI	Confidence interval
EMM	Estimated marginal mean
IQR	Interquartile range
MRI	Magnetic resonance imaging
SD	Standard deviation
TSE	Turbo Spin Echo
