# Supplementary material for: Quantitative 0.55 T MRI of the Infraclavicular Brachial Plexus: Topography and Neurovascular Morphometry Relevant to Infraclavicular Block
Source: Diagnostics (Basel). 2026 Oct 8;16(19):3247. doi: 10.3390/diagnostics16193247 (PMC13664571; doi:10.3390/diagnostics16193247)
Supplement: Supplementary file 1 [file diagnostics-16-03247-s001.zip › diagnostics-4578710-supplementary.pdf]

Supplementary Materials

Quantitative 0.55 T MRI of the Infraclavicular Brachial Plexus: Topography and Neurovascular Morphometry Relevant to Infraclavicular Block

Petar-Preslav Petrov [1,*], Delyan Dimitrov [2], Tanya Kitova [3], Pavel Timonov [4], Antoaneta Fasova [1], Darina Barbutska [1], Nikoleta Dimitrova [5,6], Yasen Getsov [7] and Milena Sandeva [3,8]

The supplementary tables provide the detailed topographic, descriptive, primary mixed-effects, evaluability, and exploratory height-adjusted sensitivity results supporting the main manuscript. All clock-face positions use the anatomically standardized orientation 12 = anterior, 3 = medial, 6 = posterior, and 9 = lateral.

Table S1. Right- and left-sided topographic distribution of the brachial plexus cords and axillary vein relative to the axillary artery.

Structure	Clock position	Right, n/N (%) [95% CI]	Left, n/N (%) [95% CI]
LC	1	3/50 (6.0%) [2.1–16.2]	2/50 (4.0%) [1.1–13.5]
LC	2	0/50 (0.0%) [0–7.1]	2/50 (4.0%) [1.1–13.5]
LC	7	1/50 (2.0%) [0.4–10.5]	0/50 (0.0%) [0–7.1]
LC	9	0/50 (0.0%) [0–7.1]	1/50 (2.0%) [0.4–10.5]
LC	10	7/50 (14.0%) [7.0–26.2]	3/50 (6.0%) [2.1–16.2]
LC	11	33/50 (66.0%) [52.2–77.6]	29/50 (58.0%) [44.2–70.6]
LC	12	6/50 (12.0%) [5.6–23.8]	13/50 (26.0%) [15.9–39.6]
MC	2	1/50 (2.0%) [0.4–10.5]	0/50 (0.0%) [0–7.1]
MC	3	18/50 (36.0%) [24.1–49.9]	23/50 (46.0%) [33.0–59.6]
MC	4	14/50 (28.0%) [17.5–41.7]	20/50 (40.0%) [27.6–53.8]
MC	5	13/50 (26.0%) [15.9–39.6]	4/50 (8.0%) [3.2–18.8]
MC	6	3/50 (6.0%) [2.1–16.2]	2/50 (4.0%) [1.1–13.5]
MC	7	1/50 (2.0%) [0.4–10.5]	1/50 (2.0%) [0.4–10.5]
PC	3	2/50 (4.0%) [1.1–13.5]	0/50 (0.0%) [0–7.1]
PC	4	3/50 (6.0%) [2.1–16.2]	4/50 (8.0%) [3.2–18.8]
PC	5	12/50 (24.0%) [14.3–37.4]	14/50 (28.0%) [17.5–41.7]
PC	6	22/50 (44.0%) [31.2–57.7]	18/50 (36.0%) [24.1–49.9]
PC	7	9/50 (18.0%) [9.8–30.8]	12/50 (24.0%) [14.3–37.4]
PC	8	2/50 (4.0%) [1.1–13.5]	2/50 (4.0%) [1.1–13.5]
AV	1	1/46 (2.2%) [0.4–11.3]	1/44 (2.3%) [0.4–11.8]
AV	2	33/46 (71.7%) [57.5–82.7]	31/44 (70.5%) [55.8–81.8]
AV	3	9/46 (19.6%) [10.7–33.2]	12/44 (27.3%) [16.3–41.8]
AV	4	2/46 (4.3%) [1.2–14.5]	0/44 (0.0%) [0–8.0]
AV	5	1/46 (2.2%) [0.4–11.3]	0/44 (0.0%) [0–8.0]

LC, lateral cord; MC, medial cord; PC, posterior cord; AV, axillary vein. The denominator is structure-specific because AV topography was evaluable in 46 right-sided and 44 left-sided examinations.

Table S2. Circular characteristics of the topographic positions overall and according to side.

Structure	Side	N	Circular mean (clock position)	95% CI	R
LC	Overall	100	11.19	11.04–11.35	0.901
LC	Right	50	11.05	10.84–11.26	0.906
LC	Left	50	11.34	11.11–11.57	0.901
MC	Overall	100	3.86	3.67–4.05	0.875
MC	Right	50	4.01	3.72–4.32	0.857
MC	Left	50	3.71	3.49–3.95	0.898
PC	Overall	100	5.84	5.64–6.04	0.865
PC	Right	50	5.81	5.50–6.09	0.858
PC	Left	50	5.88	5.60–6.16	0.872
AV	Overall	90	2.27	2.16–2.40	0.953
AV	Right	46	2.30	2.13–2.50	0.939
AV	Left	44	2.25	2.11–2.39	0.969

R is the mean resultant length; values closer to 1 indicate greater angular concentration. Confidence intervals for the circular mean were obtained by bootstrap resampling.

Table S3. Monte Carlo chi-square comparison of right- and left-sided sector distributions.

Structure	N	χ^2	df	Monte Carlo p	Holm-adjusted p
LC	100	8.637	6	0.1502	0.5827
MC	100	7.633	5	0.1457	0.5827
PC	100	3.125	5	0.7316	1.0000
AV	90	3.448	4	0.5876	1.0000

No structure demonstrated a statistically significant side difference after Holm correction.

Table S4. Descriptive intercord distances according to side and sex.

A. According to side

Distance	Side	N	Mean ± SD, mm	Median (IQR), mm	Min–Max, mm
LC–MC	Right	50	12.54 ± 3.25	12.39 (4.71)	7.04–21.85
LC–MC	Left	50	10.92 ± 2.91	11.10 (4.11)	4.90–18.06
LC–PC	Right	50	11.37 ± 2.42	11.21 (2.95)	6.02–16.82
LC–PC	Left	50	10.81 ± 2.42	10.49 (3.74)	5.61–16.23
MC–PC	Right	50	8.18 ± 2.75	7.46 (3.14)	4.05–17.31
MC–PC	Left	50	8.09 ± 2.94	7.49 (2.70)	4.48–22.72

B. According to sex

Distance	Sex	N	Mean ± SD, mm	Median (IQR), mm	Min–Max, mm
LC–MC	Women	49	10.70 ± 2.83	10.45 (4.05)	5.30–17.99
LC–MC	Men	51	12.73 ± 3.20	12.60 (3.90)	4.90–21.85
LC–PC	Women	49	10.76 ± 2.09	10.62 (2.34)	5.93–16.82
LC–PC	Men	51	11.41 ± 2.69	11.62 (4.01)	5.61–16.12
MC–PC	Women	49	7.91 ± 2.45	7.41 (3.10)	4.59–15.23
MC–PC	Men	51	8.35 ± 3.17	7.69 (2.82)	4.05–22.72

Table S5. Detailed linear mixed-effects analysis of intercord distances.

A. Omnibus tests

Effect	F	df1	df2	p
Distance type	64.547	2	192.0	<0.0001
Side	3.505	1	96.0	0.0642
Sex	6.864	1	96.0	0.0102
Distance type × Sex	3.163	2	192.0	0.0445
Distance type × Side	2.596	2	192.0	0.0772
Side × Sex	0.065	1	96.0	0.7995

Effect	F	df1	df2	p
Distance type × Side × Sex	0.003	2	192.0	0.9965

B. Estimated marginal means

Distance	EMM, mm	SE	95% CI
LC–MC	11.71	0.276	11.17–12.25
LC–PC	11.08	0.276	10.54–11.63
MC–PC	8.13	0.276	7.59–8.67

C. Pairwise comparisons with Holm correction

Comparison	Difference, mm	SE	t	df	Holm-adjusted p
LC–MC − LC–PC	+0.629	0.337	1.87	192	0.0632
LC–MC − MC–PC	+3.583	0.337	10.64	192	<0.0001
LC–PC − MC–PC	+2.954	0.337	8.77	192	<0.0001

D. Sex contrasts within each distance type

Distance	Men − Women, mm	p	Holm-adjusted p	Interpretation
LC–MC	+1.994	<0.001	≈0.0012	Significant
LC–PC	+0.645	0.2429	0.4858	Not significant
MC–PC	+0.432	0.4339	0.4858	Not significant

Because the distance type × sex interaction was significant, sex contrasts within distance type are directly relevant to interpretation. The participant-specific random intercept accounts for the three correlated distance outcomes contributed by each participant. The random-intercept ICC reported in the model characteristics quantifies within-participant correlation among the three distance-type outcomes and is not an intraobserver or interobserver reliability coefficient.

E. Model characteristics

Measurements	Participants	Random-intercept ICC	Marginal R^2	Conditional R^2	Convergence
300	100	0.254	0.290	0.470	Yes

F. Estimated marginal means according to sex × side

Distance	Sex	Side	EMM, mm	95% CI
LC–MC	Women	Right	11.47	10.36–12.57
LC–MC	Women	Left	9.96	8.88–11.05
LC–MC	Men	Right	13.53	12.47–14.60
LC–MC	Men	Left	11.88	10.80–12.97
LC–PC	Women	Right	10.98	9.88–12.09
LC–PC	Women	Left	10.54	9.45–11.62
LC–PC	Men	Right	11.72	10.66–12.79
LC–PC	Men	Left	11.09	10.00–12.17
MC–PC	Women	Right	7.89	6.78–8.99
MC–PC	Women	Left	7.94	6.85–9.02
MC–PC	Men	Right	8.45	7.39–9.51
MC–PC	Men	Left	8.24	7.16–9.33

Table S6. Detailed linear mixed-effects analysis of cord-to-axillary-artery distances.

A. Omnibus tests

Effect	F	df1	df2	p
Distance type	13.449	2	192.0	<0.0001
Side	0.087	1	96.0	0.7682
Sex	11.844	1	96.0	0.0009

Effect	F	df1	df2	p
Distance type × Side	0.625	2	192.0	0.5364
Distance type × Sex	0.013	2	192.0	0.9872
Side × Sex	0.005	1	96.0	0.9439
Distance type × Side × Sex	1.215	2	192.0	0.2990

B. Estimated marginal means

Distance	EMM, mm	SE	95% CI
LC–AA	6.32	0.182	5.96–6.68
MC–AA	7.35	0.182	6.99–7.71
PC–AA	7.13	0.182	6.77–7.48

C. Pairwise comparisons with Holm correction

Comparison	Difference, mm	SE	t	df	Holm-adjusted p
LC–AA − MC–AA	−1.031	0.209	−4.93	192	<0.0001
LC–AA − PC–AA	−0.804	0.209	−3.85	192	0.0003
MC–AA − PC–AA	+0.227	0.209	1.09	192	0.2789

D. Principal fixed-effect estimates

Comparison	Estimate, mm	95% CI	p
Men − Women	+0.936	0.401–1.471	0.0009
Left − Right	−0.081	−0.616–0.455	0.7679
Sex × Side	−0.038	−1.109–1.032	0.9439

E. Model characteristics

Measure-ments	Participants	Random-intercept ICC	Marginal R^2	Conditional R^2	Convergence
300	100	0.340	0.119	0.418	Yes

F. Estimated marginal means according to sex × side

Distance	Sex	Side	EMM, mm	95% CI
LC–AA	Women	Right	5.82	5.09–6.55
LC–AA	Men	Right	7.01	6.31–7.71
LC–AA	Women	Left	5.85	5.14–6.57
LC–AA	Men	Left	6.61	5.89–7.32
MC–AA	Women	Right	6.92	6.19–7.65
MC–AA	Men	Right	8.03	7.33–8.74
MC–AA	Women	Left	6.87	6.15–7.58
MC–AA	Men	Left	7.59	6.88–8.31
PC–AA	Women	Right	6.75	6.02–7.49
PC–AA	Men	Right	7.31	6.61–8.01
PC–AA	Women	Left	6.58	5.87–7.30
PC–AA	Men	Left	7.85	7.14–8.57

Table S7. Detailed descriptive and linear mixed-effects analysis of cord-to-axillary-vein distances.

A. Descriptive statistics according to side

Distance	Side	N	Mean ± SD, mm	Median (IQR), mm	Min–Max, mm
LC–AV	Right	45	17.84 ± 5.26	17.15 (8.71)	5.94–28.12
LC–AV	Left	47	16.20 ± 4.67	15.14 (5.49)	2.15–27.52
MC–AV	Right	45	9.54 ± 2.74	9.86 (3.41)	4.44–15.65
MC–AV	Left	47	9.68 ± 3.08	8.86 (3.94)	5.31–19.47
PC–AV	Right	45	16.79 ± 3.49	16.53 (4.42)	9.90–24.59

Distance	Side	N	Mean ± SD, mm	Median (IQR), mm	Min–Max, mm
PC–AV	Left	47	16.30 ± 4.22	16.36 (5.50)	8.61–28.60

B. Descriptive statistics according to sex

Distance	Sex	N	Mean ± SD, mm	Median (IQR), mm	Min–Max, mm
LC–AV	Women	46	16.03 ± 4.44	15.03 (5.87)	5.94–28.12
LC–AV	Men	46	17.98 ± 5.40	17.15 (7.92)	2.15–27.88
MC–AV	Women	46	8.88 ± 2.38	8.66 (3.47)	4.44–14.64
MC–AV	Men	46	10.34 ± 3.21	9.95 (4.29)	5.45–19.47
PC–AV	Women	46	15.77 ± 3.74	15.67 (4.40)	9.90–28.60
PC–AV	Men	46	17.30 ± 3.89	16.84 (5.10)	8.61–27.14

C. Omnibus tests

Effect	F	df1	df2	p
Distance type	198.695	2	176	<0.0001
Sex	6.479	1	88	0.0127
Side	1.121	1	88	0.2926
Distance type × Sex	0.265	2	176	0.7675
Distance type × Side	2.382	2	176	0.0953
Sex × Side	2.146	1	88	0.1465
Distance type × Sex × Side	1.445	2	176	0.2386

D. Estimated marginal means

Distance	EMM, mm	SE	95% CI
LC–AV	17.04	0.409	16.24–17.90
MC–AV	9.62	0.409	8.81–10.40
PC–AV	16.55	0.409	15.75–17.40

E. Pairwise comparisons with Holm correction

Comparison	Difference, mm	SE	t	df	Holm-adjusted p
LC–AV − MC–AV	+7.428	0.417	17.82	176	<0.0001
LC–AV − PC–AV	+0.490	0.417	1.18	176	0.2408
MC–AV − PC–AV	−6.937	0.417	−16.65	176	<0.0001

F. Principal fixed-effect estimates

Comparison	Estimate, mm	95% CI	p
Men − Women	+1.684	0.381–2.987	0.0127
Left − Right	−0.701	−2.004–0.602	0.2923
Sex × Side	−1.939	−4.545–0.668	0.1465

G. Model characteristics

Measurements	Participants	Random-intercept ICC	Marginal R^2	Conditional R^2	Convergence
276	92	0.481	0.453	0.716	Yes

H. Estimated marginal means according to sex × side

Distance	Sex	Side	EMM, mm	SE	95% CI
LC–AV	Women	Right	15.99	0.818	14.38–17.60
LC–AV	Women	Left	16.06	0.818	14.45–17.70
LC–AV	Men	Right	19.78	0.836	18.13–21.40
LC–AV	Men	Left	16.34	0.801	14.76–17.90
MC–AV	Women	Right	8.64	0.818	7.02–10.20
MC–AV	Women	Left	9.13	0.818	7.52–10.70
MC–AV	Men	Right	10.49	0.836	8.84–12.10
MC–AV	Men	Left	10.21	0.801	8.63–11.80

Distance	Sex	Side	EMM, mm	SE	95% CI
PC–AV	Women	Right	15.66	0.818	14.04–17.30
PC–AV	Women	Left	15.89	0.818	14.28–17.50
PC–AV	Men	Right	17.97	0.836	16.32–19.60
PC–AV	Men	Left	16.69	0.801	15.11–18.30

The primary relationship was MC–AV < LC–AV ≈ PC–AV.

Table S8. Exploratory height-adjusted sensitivity analyses of the three morphometric distance families.

Distance family	Observations / participants	Sex	Height	Side	Distance type × Sex
Intercord	300 / 100	F(1,95)=1.058; p=0.3063	F(1,95)=11.882; p=0.0008	F(1,95)=3.828; p=0.0533	F(2,192)=3.163; p=0.0445
Cord-to-AA	300 / 100	F(1,95)=4.717; p=0.0323	F(1,95)=5.652; p=0.0194	F(1,95)=0.084; p=0.7727	F(2,192)=0.013; p=0.9872
Cord-to-AV	276 / 92	F(1,87)=2.176; p=0.1438	F(1,87)=2.980; p=0.0878	F(1,87)=1.097; p=0.2978	F(2,176)=0.265; p=0.7675

Height was entered as an additional continuous covariate (main effect only) while retaining the original distance type, sex, side, interaction terms, and participant random intercept. Distance type remained significant in all three sensitivity models (all p<0.0001). Other interactions were non-significant except the intercord distance type × sex interaction shown above. These analyses were exploratory and did not replace the primary models.

Abbreviations: AA, axillary artery; AV, axillary vein; CI, confidence interval; EMM, estimated marginal mean; ICC, intraclass correlation coefficient (in model-characteristics tables, the random-intercept within-participant correlation, not observer reproducibility); IQR, interquartile range; LC, lateral cord; MC, medial cord; PC, posterior cord; SD, standard deviation; SE, standard error.
